# A brief intervention to discontinue inappropriate z-hypnotics use by older patients in primary care: a randomised controlled trial

**DOI:** 10.1186/s12875-026-03212-w

**Published:** 2026-02-13

**Authors:** Tahreem Ghazal Siddiqui, Tone Breines Simonsen, Maria Torheim Bjelkarøy, Maria Lie Selle, Christofer Lundqvist

**Affiliations:** 1 Health Services Research Unit (HØKH), Akershus University Hospital, Lørenskog, Norway; 2 Institute of Clinical medicine, Faculty of medicine, University of Oslo, Oslo, Norway

**Keywords:** Hypnotics, Deprescription, Brief intervention, Older adults, General practitioner, Randomised controlled trial

## Abstract

**Introduction:**

Healthcare guidelines advise that z-hypnotics should not be used long-term in older patients. However, inappropriate use of these medications, characterised by prolonged use at high doses, remains common. This study aimed to evaluate the effect of a brief intervention (BI) conducted by general practitioners (GPs) for terminating inappropriate use of z-hypnotics, compared to business as usual (BAU – control group).

**Methods:**

This study was a double-blind, randomised controlled trial comparing BI conducted by trained GPs to BAU at baseline and at a six-week follow-up. Both intention-to-treat (ITT) and per-protocol (PP) analyses were performed, utilising t-tests and Fisher’s exact tests. The predefined primary outcome was the proportion of participants with no inappropriate z-hypnotic use (defined as use for four weeks or more, or three times per week or more). Secondary outcomes included sleep problems, measured by the Global Sleep Assessment Scale, visual analogue scale for (VAS) pain intensity, and cognitive function, measured by the Montreal Cognitive Assessment (MOCA).

**Results:**

Both study arms reduced inappropriate use and improved usage patterns (*n* = 45). No difference was found in the ITT analysis of BI (*n* = 23) and BAU (*n* = 22) at six-week follow-up (no inappropriate use BAU = 71% and BI = 57%, *p* = 0.51). There were no significant differences between the BI and BAU groups in cognitive function (MOCA) (mean (SD) BI: 18.12 (2.15); BAU: 17.61 (2.89); *p* = 0.56), and VAS pain intensity (mean (SD) BI: 1.67(1.96); BAU: 1.75(2.05); *p* = 0.90). There was no significant difference in insomnia symptoms at six-week follow-up (proportion of insomnia BAU = 9.1% and BI = 8.7%, *p*-value = 1.00). The PP analysis showed comparable results.

**Conclusion:**

While many patients reduced their use of z-hypnotics, there was no significant difference in inappropriate use between the BI and BAU groups. The main limitation of the study was that it became underpowered. More studies are needed with a larger sample size.

**Trial registration:**

NCT06032715

**Supplementary Information:**

The online version contains supplementary material available at 10.1186/s12875-026-03212-w.

## Background

Z-hypnotics are frequently prescribed to treat insomnia by enhancing the effects of gamma-aminobutyric acid (GABA), the primary inhibitory neurotransmitter in the central nervous system, thereby producing a sedative effect [1]. Several guidelines and recommendations regarding the use of z-hypnotics in adults over 60 years of age suggest avoiding inappropriate use [2–4] as well as concurrent use with other central nervous system depressants (CNSDs), including opioids, analgesics, and benzodiazepines [2–6]. Inappropriate medication use in older adults, is defined as drugs that pose more risks than benefits to the patients [2]. Z-hypnotics are recommended to be used for short periods in older adults (<2–4 weeks) [4]. However, previous studies show that the prevalence of z-hypnotic use among adults over the age of 60 varies by country, ranging from approximately 8% to 25% [7–9]. In Norway, as many as 65% of those using z-hypnotics use them inappropriately [10]. In our previous study, 16% of older patients in a general practitioner (GP) population used z-hypnotics, and among them, 63% inappropriately [11]. A Norwegian study examined the prevalence of diagnosed sleep disorders and z-hypnotic prescriptions in primary healthcare. Among older patients (aged 60 to 90 years), the East Norway region exhibited the highest prevalence of sleep disorders (ranging from 7% to 13%) and the highest prevalence of z-hypnotic prescriptions (ranging from 13% to 25%) compared to other regions in Norway. These patients were twice as likely to use z-hypnotics as they were to be diagnosed with a sleep disorder [8], suggesting potential use beyond clinical indications.

The inappropriate use of z-hypnotics, also known as z-drugs, includes agents such as zolpidem and zopiclone [4], poses a risk for falls in older adults [4, 12]. A combination of z-hypnotics with other CNSDs exposes patients to a heightened risk of other adverse events and reactions, which may include increased drug toxicity and interactions, potentially leading to drug-induced delirium, overdose, decreased respiratory function, and cognitive impairment [13–16]. Older patients with prolonged z-hypnotics and other CNSD use have been found to have lower quality of life [17], more multimorbidity [18], increased risk of falls [12], and higher mortality compared to non-users [19, 20]. Studies also show that dependence and substance use disorders may occur in older patients using z-hypnotics [21, 22]. The severity of dependence scale (SDS) is a validated scale that can detect the severity of psychological dependence and thus may be used to assess dependence-like z-hypnotics use [22].

There is currently no consensus on the best approach to deprescribing z-hypnotics in older patients. Both patients and physicians find it challenging to communicate about medications [23, 24], especially when the patient has reduced cognitive function or comorbid conditions [25]. A systematic review found that interventions aimed at discontinuing benzodiazepines and z-hypnotics often involved gradual dose reduction combined with psychological therapy or psychoeducation [26]. Another systematic review suggested that short interventions may be effective in reducing the use of these medications [27]. Given the lack of consensus on how to discontinue z-hypnotics in older patients and the absence of individualised intervention studies, there is a need to develop and test systematic strategies for discontinuing z-hypnotic use in this population.

Brief intervention (BI) is a specific approach provided by professionals based on principles of simple advice or minimal interventions aimed at changing behaviours associated with substance use disorders [28]. A BI typically involves screening followed by offering patients tailored information about their substance use and a discontinuation plan that addresses possible difficulties and benefits [29]. The effectiveness of BI has been demonstrated in addressing various unhealthy behaviours such as substance use disorder, smoking, diet change, cholesterol control, and reducing painkiller use across different healthcare settings [28, 30–32].

This study aims to evaluate the effect of BI performed by GPs compared to business as usual (BAU) (control group) in terminating inappropriate z-hypnotic use among older patients in a randomised controlled trial (RCT).

## Methods

### Design

**Fig. 1:**
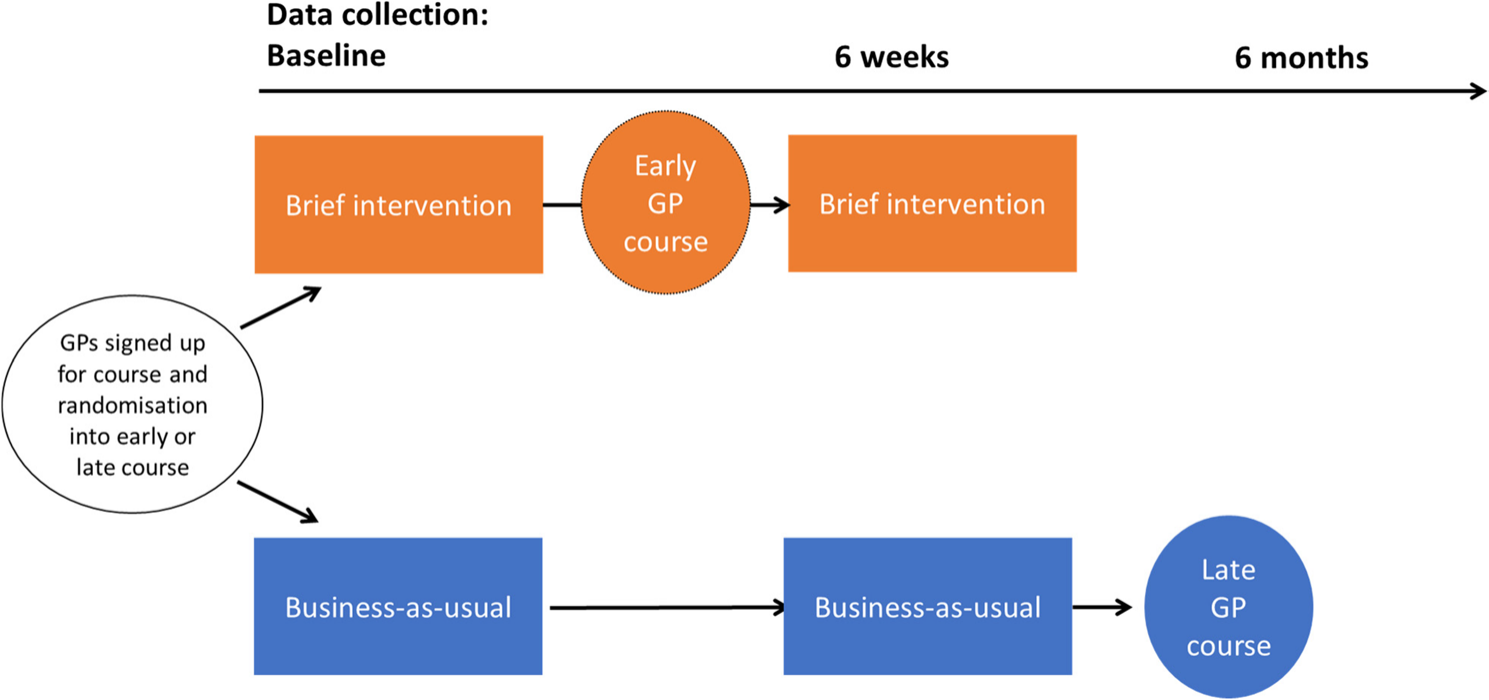
Study design, data collection, GP participation, and randomisation. Footnote: Screening of patients after general practitioners (GPs) sign up for the course, randomising GP clusters to early or late course. Early courses receive the brief intervention (BI), late course is the control group (business as usual – BAU (control group)). Data collection at baseline before intervention and six weeks of follow-up

We conducted a double-blind RCT. The period for the RCT was from October 2023 until May 2024. The data collection was ongoing until December 2024. We recruited patients from primary health care, through the GPs’ patient lists, after the GPs signed up for a course about older adults and sleep. The GPs were randomised by cluster of GP surgeries. We used the Medical Research Council (MRC) framework for complex interventions [33–35] to develop and evaluate the BI. The MRC framework involved the development of the BI tool through the use of previous literature on BI for central nervous system depressant medications, including our own studies. A pilot study [36] and regular meetings to collect feedback from user and expert group panels were also conducted in this process. The project protocol has been published separately; for more details, see [37]. The study design is illustrated in Fig. 1.

### Settings, participants and data collection

#### General practitioner

**Fig. 2:**
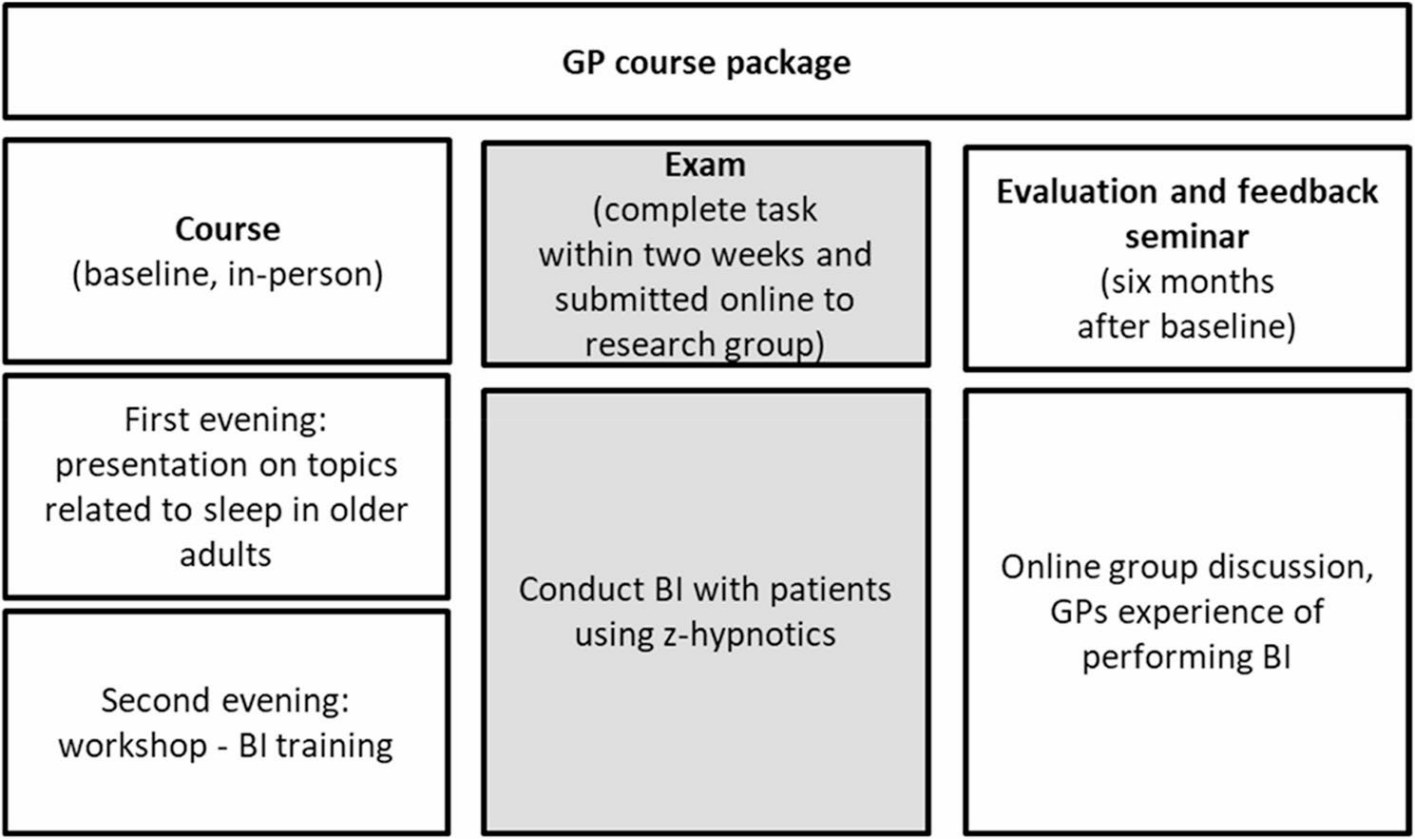
Footnote: The course included two evenings with lectures and BI training, followed by the exam, where the GPs conducted BI on their patients. We also conducted online meetings for feedback after the testing of BI

This study was set in the primary care in the catchment area of Akershus University Hospital in the south-east of Norway. In Norway, all citizens are assigned a GP. The aim was therefore to train the GPs to perform the study intervention with their patients. The research group invited GPs to participate in a clinical course about older adults and treatment guidelines for sleep disorders (approved by the Norwegian Medical Association of general practice, 15 credits), by sending an invitation to 126 GP practices. GPs were eligible if they were in the catchment area of our hospital. In addition, the invitation was sent to the municipal medical officer, asking them to invite their GP practices to the course. Forty (19 withdrew) GPs signed up for the course during the summer of 2023. In Norway, all citizens are assigned a GP, and as part of the course, the GPs consented to provide the researchers with lists of names, addresses, and phone numbers of their patients aged 60 or above. No medical or other patient-related information was given. The GPs had 2 patients each (median, range 0–5), based on a preceding patient screening questionnaire through which consenting patients reporting use of z-hypnotics were selected [11]. As part of the in-person course, we incorporated training on how to conduct a BI for inappropriate z-hypnotics use (see Fig. 2). The intervention was part of the practical exam, where the GPs would conduct BI on their own patients, assigned by us through screening of their patient list. We used a block design where the GPs were randomised to take part in either the first block (early course group) or in the second block six months later (late course group). The early course group received BI training first and conducted the BI on their patients, and the late course group acted as the BAU (control) group. Data collection time points reported in this article are baseline before intervention and six weeks after BI/BAU.

#### Patients

**Fig. 3:**
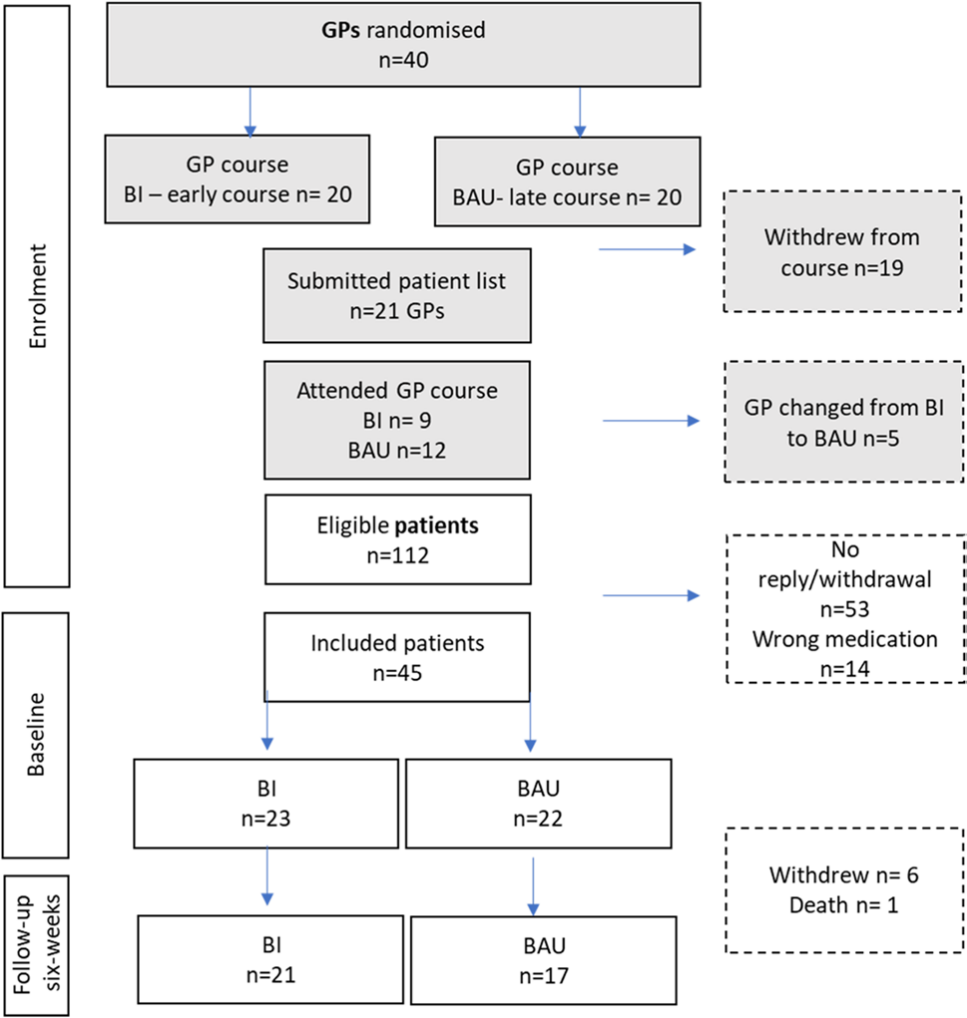
Flowchart. BI=brief intervention, BAU=business-as-usual (control group), GP=general practitioner

After the GPs signed up for the course, but before the course started, we recruited the patients prospectively by contacting them through SMS using the patient contact lists. They received a link to an online information letter, a digital consent form, and an online questionnaire about sleep problems and z-hypnotics use. Providing the return of a valid digital consent form, we then screened the patients for z-hypnotics use based on their self-reports [11], and if eligible and consented for recontact, they were contacted and invited to the RCT. The inclusion criteria were age 60 years and above and self-reporting use of z-hypnotics for ≥four weeks; the cut-off was based on our previous study [22]. The patient inclusion was thus based on screening before the RCT [11]. Figure 3 shows the flow of participants through the study.

### Measurements

#### Primary outcome

The proportion of participants with inappropriate z-hypnotics use (using z-hypnotics for the last ≥four weeks, three times per week) was compared between the BI and BAU groups at six weeks after baseline.

#### Secondary outcomes

Secondary outcomes, including sleep, pain, and cognitive function, were assessed six weeks after baseline, in BI versus BAU. In addition, a between-group and within-group comparison of change in inappropriate use compared to baseline was performed.

#### Z-hypnotics use and other medications

GPs in Norway have information about their patients’ prescribed medications, including prescriptions from hospitals. However, the patients can give a more accurate description of their intake, as the prescriptions of the GP or in the prescription registry do not always match what patients are taking. Therefore, we primarily used online questionnaires to ask the patients about their frequency of use and dose over the past four weeks at baseline and at six-week follow-up. For those who had unclear or missing answers on the questionnaire, we supplemented with phone interview data on medication use. We also asked the GPs at the time of the intervention to report their patients’ z-hypnotics brand name and dosage. To avoid selection bias, the GPs did not recruit their own patients, but later confirmed whether the patient had a prescription for z-hypnotics or not, including zolpidem and zopiclone, and other non-benzodiazepines purchased abroad (not available in Norway). Inappropriate use was defined for this study as the length of z-hypnotics used z-hypnotics for the last ≥four weeks, three times per week), versus use < three times per week over the same period.

#### Demographic information

An online questionnaire was developed for this study, we included questions about age (years), gender (male, female, other), education (primary, secondary, high school, or university level), and income (categories of NOK per year) (for more details see supplement 1).

#### Cognitive function *-* Montreal cognitive assessment

We used the visually impaired version of the Montreal cognitive assessment (MOCA) [38], with Norwegian translation from version (8.1 and 8.3), to assess the global cognitive function among the participants. The condensed version was scored from 0 to 22, where lower scores show mild cognitive impairment [38].

#### Pain - visual analogue scale

We used the visual analogue scale (VAS) (0–10 cm, higher number indicated more pain intensity) [39] and one question related to whether patients experienced pain (yes/no). This has previously been validated in older patients [40].

#### Sleep - global sleep assessment questionnaire

Global sleep assessment questionnaire (GSAQ) is an 11-item questionnaire that screens for sleep disorder categories, including: insomnia, sleep apnoea, restless leg syndrome, parasomnia, and hypersomnia. In addition, secondary causes of sleep disorders, including anxiety/depression, pain, physical problems, worrying, and medication use, are recorded. The response to each question is noted as never, sometimes, usually, or always. The GSAQ sleep disorders are categorised based on the original scoring [41] and are labelled as a positive diagnosis if item responses include usually or always, and negative for never or sometimes. The diagnostic categories are presented descriptively. A diagnosis of insomnia was further used in a secondary analysis.

### Intervention

Steps 1–6 describing the intervention and interaction between GP and patient:

1. *Assessment*: The GP uses open questions (e.g., how is your sleep quality? ), then screens the patient using the severity of dependence scale (SDS) [22] to assess whether the patient is at risk of dependence on z-hypnotics.
2. *Feedback*: The patient gets feedback based on the SDS score (if a score ≥ 6, see*). The GP gives information about who is generally found to be at risk risky use and how that is related to the patient.
3. *Advice*: The GPs give advice and motivate the patient to change the risky use, tailoring it to the patient’s situation. Importantly, challenges such as temporary withdrawal effects and rebound insomnia are discussed in relation to loss vs. gains.
4. *Options*: The patient and GP discuss options, including treatment goals (e.g., stopping, reducing, waiting, doing a short follow-up, or re-evaluating later). This step is based on a collaboration between the GP and patients.
5. *Reflection*: The GP and patient discuss responsibilities, readiness to change, options for support, reflection on goals, and planning for treatment.
6. *Treatment plan*: The GP and patient sum up the treatment plan for home.

*If the patients have a SDS score < 6: the GP completes steps two and three without discussing specific treatment plans in steps four through five,* and summaries in step six by explaining that the patient is not yet “at risk”-user but could fall into this category later.*

### Randomisation

The statistician (MLS) received an anonymised list with ID numbers for each GP and the surgery to which they belonged. MLS performed cluster randomisation of the GPs with their patients. The patient was thus randomised either to a GP participating in an earlier course or to a GP receiving the course later. The GPs were clustered because some GPs belonged to the same GP-surgeries. This was done to avoid “contamination of information” between the intervention and control arm. GPs were randomised to the early course (BI arm) or the late course (BAU control arm) (Fig. 2).

### Blinding

The study coordinator (TBS) managed the data collection, and the assessor (TGS, MTB, and CL) did not know which patients belonged to the intervention or control group at baseline and follow-up. The statistician (MLS) was blinded to which study arm the patients belonged to when analysing the data. The GPs in the BAU (control) group and their patients were “masked” as they did not know that the brief intervention was a part of the study. The GPs were only informed that they would participate in a course about sleep in older adults in the late course (Fig. 1) during spring 2024. The BI was a part of the GPs coursework to get credits. Likewise, the patients consented to participating in a study to evaluate sleep and optimise their medication and were “masked” to the brief intervention until their GPs had attended the course.

### Sample size

The primary outcome in this study is the proportion of patients without inappropriate use of z-hypnotics (last ≥four weeks, three times per week) We assumed that the proportion of patients with no inappropriate use (< three times per week) six weeks after baseline would be 30% in the BI and 2% in the BAU group. To detect a difference in this primary outcome between the groups at 5% significance level and with a power of 80% using a z-test for proportions, the estimated number of patients needed was 26 in each group, based on previous experience with a similar study [32].

### Statical analyses

We analysed the data using the Intention-to-treat (ITT) principle as this is the standard for RCTs. However, the ITT was clearly compromised early on, as some GPs randomised to the BI arm dropped out of the early course, and thus their patients did not receive the BI. We have therefore also included the per-protocol (PP) analyses.

We examined one primary outcome – the proportion of participants without inappropriate z-hypnotics use and three secondary outcomes – sleep disorders, pain intensity, and cognitive function, comparing them between the arms six weeks after baseline. The outcomes were originally planned to be compared between BI and BAU groups by a z-test for proportions for the primary outcome and an independent-samples t-test for secondary outcomes. Due to the small sample size, we used Fisher’s exact test for the primary outcome and for insomnia diagnosis. Seven patients had missing outcomes at the six-week follow-up and were therefore removed from the tests. Within-group change from baseline to six weeks was estimated for the primary outcome using McNemar’s test for paired proportions.

In addition, we performed a within and between group comparison of change using a mixed logistic regression model (binary outcome: no inappropriate use was successful), with fixed effects of age, gender, treatment group (BAU and BI), time points (baseline and six weeks), and interaction between group and time. The model had a random intercept for patient ID, nested within GP ID. A linear mixed effects regression model was estimated for the secondary outcomes (MOCA, pain VAS), with the same fixed and random effects as the logistic model. Since the estimated GP-level variance was approximately zero in the models for the primary outcome and cognitive function, this level was taken out of the models. The McNemar test for paired proportions was used for insomnia diagnosis. All tests were two-sided with a 5% significance level, and tests were performed using R version 4.4.2.

### Ethical considerations

This study complies with the Declaration of Helsinki and was performed according to ethics committee approval, and all participants provided written informed consent. Further, the study procedures, data collection, and storage were also approved by the Akershus University Hospital data protection officer and the Regional Committee for Medical and Health Research Ethics (reference: 151846/556653**)**. Written informed consent was obtained from the participants. The trial was registered on 17th Aug 2023 at clinicaltrials.gov (NCT06032715).

## Results

This section presents results analysed according to the ITT principle unless otherwise stated. Relevant results from the corresponding PP analyses are presented in the tables with ITT. The descriptive statistics of business-as-usual versus brief intervention arms (ITT) are presented in Table 1.

**Table 1:** Descriptive statistics of business-as-usual versus brief intervention arms (ITT)

Variables (8)		*BAU* (*n* = 22)	*BI* (*n* = 23)
*Age at baseline*, *mean (SD)*		70 (6.26)	69 (6.13)
*Education* *(n)*	Primary school	2	7
	High school	10	9
	University	10	7
*Gender* *(n)*	Females	16	15
	Males	6	8
*Income household* *(n)*	≤ 349.000–749.000 NOK	10	14
	≥ 750.000–2 million NOK	9	8
	Missing	3	1
*Cognitive function*, *mean (SD)*	Moca baseline	18.4 (2.25)	17.8 (2.84)
	Moca six-weeks	18.1 (2.15)	17.6 (2.89)
*Pain baseline* *(n)*	No	6	9
	Yes	15	14
	VAS scale, mean (SD)	3.4 (2.58)	2.7 (2.67)
*Pain 6 weeks* *(n)*	No	8	10
	Yes	8	11
	VAS scale, mean (SD)	1.75 (2.05)	1.67 (1.96)
GSAQ screening diagnoses positive (n) *	Insomnia	1	3
	Obstructive sleep apnoea	6	10
	Shift work/parasomnia/hypersomnia restless leg syndrome	0	0
	Secondary causes of sleep disorders	0	3
GSAQ six-week *screening diagnoses positive (n)*	Insomnia	2	2
	Obstructive sleep apnoea	2	6
	Shift work/ parasomnia/ restless leg syndrome	0	0
	Hypersomnia	0	1
	Secondary causes of sleep disorders	1	0

*Footnote*: *Pain and GSAQ missing=1, *SD* standard deviation, *ITT *intention-to-treat, *BI *brief intervention, *BAU *Business-as-usual

### Descriptives

**Fig. 4:**
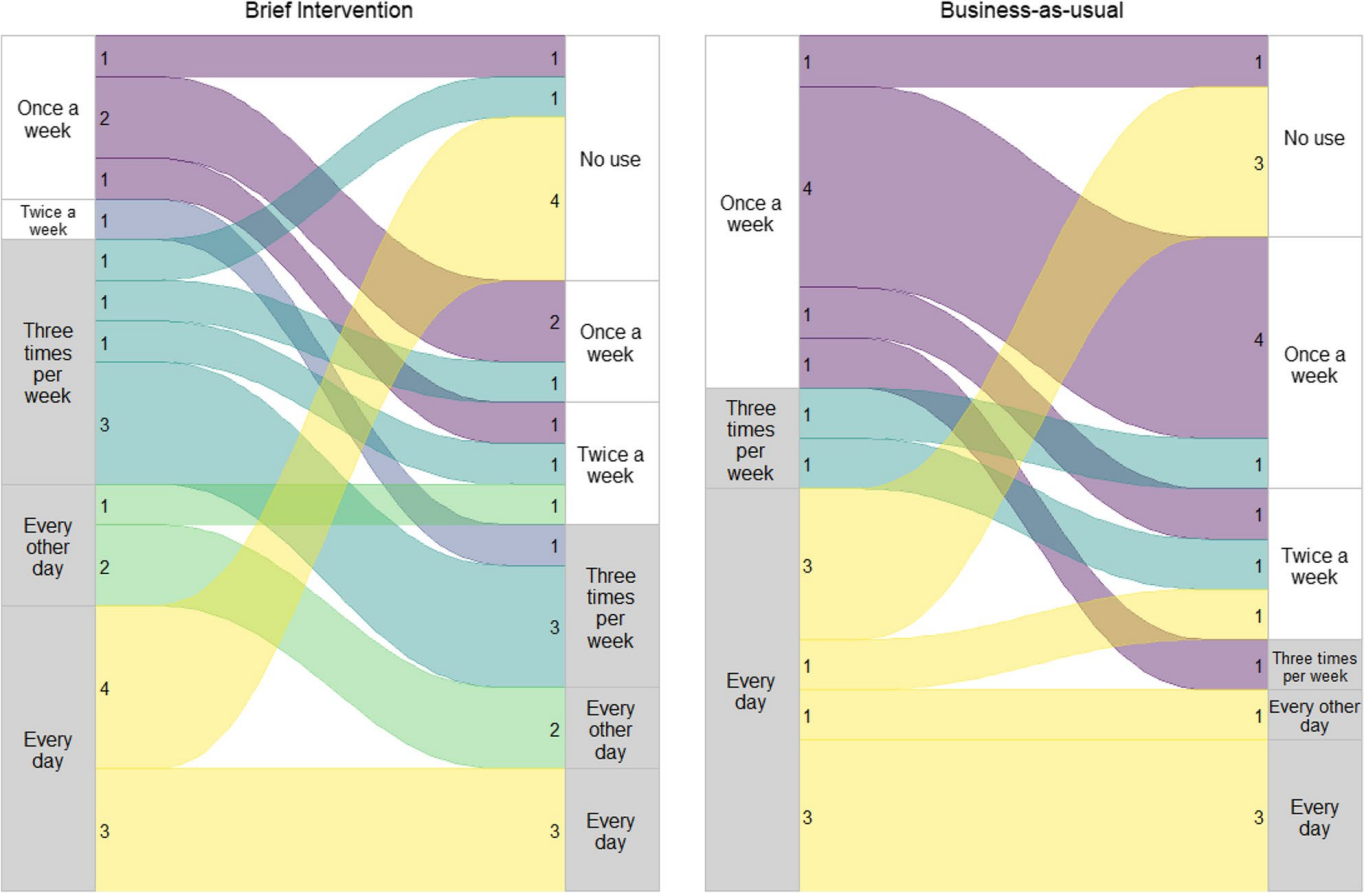
Sankey plot of frequency of z-hypnotics use in the BAU vs the BI arms. *Absolute numbers listed for each group; frequency of z-hypnotics use in the two groups. Grey bars on the side show the cut-off for inappropriate use

At baseline, the BI group consisted of 23 patients, of whom 21,7% had no inappropriate use. At six weeks follow-up, this had increased to 57.1%. The BAU group had 22 patients, of whom 40.9% had no inappropriate use at baseline. This had increased to 70,6% at six-week follow-up. The distribution of z-hypnotic use is shown in Fig. 4. In the PP analysis, 16 patients were included in the BI group at baseline, where 12,5% had no inappropriate use, which increased to 50% at six weeks. In the BAU group, 29 patients were included at baseline, where 41,4% had no inappropriate use, which increased to 72,7% at six weeks.

The type of medication used by the participants was: Zopiclone (*n* = 37), Zolpidem (*n* = 5), and non-benzodiazepines used for insomnia (*n* = 3) (not available in Norway, purchased abroad).

### Primary outcome

The main analysis for the primary outcome showed no significant difference between the BI and BAU group at six-week follow-up (proportion of no inappropriate use and BI = 57.1% and BAU = 70.6%, *p*-value = 0.51).

### Secondary outcomes

After six-week follow-up, there were no significant differences between the BI and BAU groups in cognitive function (short MOCA score) (mean (SD) BI: 18.12 (2.15); BAU: 17.61 (2.89); *p* = 0.56), and VAS pain intensity (mean (SD) BI: 1.67(1.96); BAU: 1.75(2.05); *p* = 0.90). There was no significant difference between groups with respect to insomnia symptoms at the six-week follow-up (the proportions of insomnia symptoms in the BAU and BI groups were 9.1% and 8.7%, respectively; *p* = 1.00). The PP analysis showed comparable results.

### Logistic mixed model regressions

The within-group analysis showed that across both arms, there was a significant increase in patients with appropriate use, from 31.1% at baseline to 63.2% at six weeks (*p* = 0.006). Within the BI, this increase in proportions was from 21.7% at baseline to 57.1% at six weeks with a significant difference (*p* = 0.046). Within the BAU arm, the same increase in proportions was from 40.9% at baseline to 70.6% at six weeks, with no significant difference (*p* = 0.13). PP analysis showed comparable results.

We examined the odds ratio for success (no inappropriate z-hypnotics use) from baseline to six-week follow-up, adjusting for sex and age in a mixed logistic regression model. For the BI group, the odds of no inappropriate use were 8.09 (95% CI: 1.34–48.73) times higher at follow-up compared to baseline, and for the BAU group, the odds were 4.02 (95% CI: 0.75–21.57) times higher at six weeks. The relative difference in odds ratio between the BI and BAU was 2.01 (95% CI: 0.22–18.27). The PP analyses showed comparable results (see Table 2).

**Table 2:** Estimated odds ratio for the primary outcome (no inappropriate use) from baseline to six weeks for BI and BAU

	ITT Estimates	PP Estimates
	OR (95 % CI Lower – upper)	OR (95 % CI Lower – upper)
BAU	4.02 (0.75: 21.57)	5,09 (0.98: 19.32)
BI	8.09 (1.34: 48.73)	10,49 (1.23: 110.95)
BI/BAU	2.01(0.22: 18.27)	2,06 (0.23: 30.96)

*Footnote*: The model had fixed effects for age, gender, group (BAU and BI), time points (baseline and six weeks), and the interaction between group and time. *OR* odds ratio, *CI* 95 % confidence interval, *BI* brief intervention, *BAU* Business-as-usual

### Linear mixed model regression

The analysis of within-group changes showed that both BI (not significant) and BAU had a small reduction in pain over time, while neither arm had significant changes in the cognitive function score. We found no significant difference in change between the groups (Table 3). Due to the small sample size, we did not estimate effects on insomnia symptoms using a logistic mixed model.

**Table 3:** Estimated coefficients for the secondary outcomes from baseline to six weeks for BI and BAU

	ITT(β)	95% CI lower - upper	PP(β)	95% CI lower -upper
Cognitive function				
Change baseline to six weeks, BAU	-0,35	-1,64: 0,95	-0,30	-1,44: 0,83
Change baseline to six weeks, BI	-0,24	-1,49: 1,01	-0,30	-1,78: 1,18
Difference in change (BI-BAU)	0,11	-1,69: 1,90	0,00	-1,87: 1,87
Pain				
Change baseline to six weeks, BAU	-1,25	-2,29: -0,22	-1,37	-2,25: -0,48
Change baseline to six weeks, BI	-0,80	-1,69: 0,10	-0,44	-1,46: 0,58
Difference in change (BI-BAU)	0,46	-0,91: 1,83	0,93	-0,42: 2,28

*Footnote*: the model had fixed effects for age, gender, group (BAU and BI), time points (baseline and six weeks), and the interaction between group and time. β =regression coefficients, *CI * 95% confidence interval, *BI *brief intervention, *BAU *Business-as-usual

## Discussion

To the best of our knowledge, this is the first study to examine a BI primarily focused on z-hypnotics. We conducted an RCT of BI carried out by trained GPs versus BAU. Unfortunately, due to circumstances beyond our control, the study became underpowered. However, our findings suggest that the proportion of patients with inappropriate z-hypnotic use did not differ statistically between the two study arms, though both arms improved. Thus, the predefined primary outcome was negative. The main secondary outcomes, cognition, pain, and sleep, also did not change significantly. The PP analyses supported the ITT findings. Patients in both groups significantly reduced their inappropriate use of z-hypnotics at the six-week follow-up compared to baseline, indicating that even simple contact with patients regarding their z-hypnotic use can help them reduce or stop inappropriate usage. When examining within-group differences, we found a significant reduction in inappropriate use from baseline to follow-up in the BI group, which was not the case for the BAU group. Previous research showed that short interventions informing older patients about risks associated with prolonged use of anxiolytics, z-hypnotics, and sedatives helped patients reduce or stop their use in the intervention group. Similarly to our results, positive effects were also shown in the control group [42–46]. In our study, we also found such an assessment effect. The reason for the reduction in the control group during the study is likely to be related to the study-related information about sleep and sleep medication provided through consent documents and questionnaires, contributing to patients reducing their medication without help from their GP.

Studies show that a long-lasting reduction of benzodiazepines, including z-hypnotics, may be achieved over a 6 to 12-month study period [42–47]. The degree of complexity and duration of the intervention methods varied in these studies and included GP consultations with information letters or booklets [42, 43], consultations with follow-up or written instructions [44, 45], or workshops for GP education [46]. In our study, we conducted a single GP consultation with a short follow-up period of six weeks without additional pre-planned contacts. Despite such limited patient contact, we observed evidence of improved z-hypnotic intake patterns in both groups, suggesting that such changes may be achievable with merely a focus on sleep medications, either through direct discussions about medications or by raising awareness of the study’s focus. Unfortunately for an RCT, such assessment effects influence the likelihood of identifying a significant difference between the active (BI) and control (BAU) groups in a small study.

In the descriptive analysis, we saw low rates of insomnia symptoms, but they were higher at baseline for the BI group. Interestingly, sleep apnoea was reduced after the intervention. This needs to be further investigated in a larger sample. Sleep disturbances can contribute to cognitive decline, potentially influencing conditions like dementia [48]. Z-hypnotics have been suggested to influence cognitive processes, and in a previous study, we found reduced cognitive function in older hospitalised patients using z-hypnotics [49]. We found no significant difference in cognitive function between the two groups at the six-week follow-up or compared to baseline despite the reduced z-hypnotics use.

Pain can disrupt sleep, while poor sleep can heighten pain sensitivity, indicating a bidirectional relationship [50]. We have previously found an association between combined use of opioids and z-hypnotics [40] and pain among the older hospitalised population [17, 22]. However, here we found no significant difference in pain despite reduced z-hypnotics use. To clarify these relationships, further studies with larger samples will be necessary. Still, while our study was small, it does not indicate significant risks of negative outcomes in sleep, pain, or cognition following the discontinuation of z-hypnotics.

### Limitations

The main limitation of the study was that it became underpowered, largely due to fewer GPs taking part than expected and to GPs dropping out before intervention had started. We also had difficulty in recruiting patients with z-hypnotics use, as some of them declined to participate after the initial screening, which can lead to selection bias. The study was blinded to the patients, GPs, and researchers. However, it is difficult to keep the patients fully blinded under a behavioural intervention and when using patient-reported data. Despite minimal patient contact, we see a possible assessment effect in the control group. We attempted to avoid suggesting to BAU patients that their medication use was inappropriate or that they should reduce their sleep medication intake; however, a focus on sleep and sleep medications was clear in consent documents, questionnaires, and tests. A regression to the mean may also be a factor leading to changes in z-hypnotics use over time. An alternative and simpler recruitment process may be to use GPs to recruit patients who use z-hypnotics more actively. However, the risk of greater selection bias would then be expected. Nevertheless, the advantage of GPs recruiting patients, in addition to simplified logistics, could be an increased response rate from patients and a more pragmatic, real-life type of study.

Unfortunately, the randomisation of GPs was not fully successful as some dropped out of participating in the training course, and therefore their patients could not participate in the study. Some of the reasons for withdrawal were due to technological challenges with the patients’ lists. Other reasons were related to sick leave, temporary absence, and distance to the course location. Five of the GPs originally assigned to BI changed their course group, and they and their patients were thus effectively moved to the control group, which was unfortunate in terms of the originally planned ITT analysis approach. In addition, the power calculations were not done with the cluster effect of the GP surgeries, but we assume this effect to be minimal [37].

This study was done in a selected GP population, as the GPs enrolled for the course themselves, and cannot necessarily be generalised to the general population. However, we have no reason to suspect that these GPs were quite different from other GPs in the area, and there is no reason to suspect differences in GP profile between the study arms. In addition, older and sicker patients might need further support in stopping their inappropriate use. The control group had more patients without inappropriate use at baseline compared to the BI group. Thus, more “heavy users” in the BI group could have made it more difficult to stop inappropriate z-hypnotic use.

In addition, even though BI is a single-time point intervention, the treatment plan has the possibility for a follow-up consultation. If the GP and the patients agreed on having further consultation or planned for a discontinuation plan for z-hypnotics after six-week follow-up, our assessment at six-week follow-up would not have detected those changes. Thus, a longer follow-up study is necessary. Moreover, barriers to implementing interventions of this nature include a lack of time, financial disincentives, and organisational challenges within the healthcare system [51, 52]. To address some of these barriers, we have taken specific measures such as keeping the BI short and making it possible to conduct during one consultation. Importantly, we plan a qualitative study of GPs’ experience with the use of the intervention.

### Strengths

The BI has several advantages. Firstly, it is easy to conduct during a single consultation, and it takes a systematic approach to reduce prolonged use of z-hypnotics and mitigate the risk of adverse events, while being open to adjusting the contents of the dialogue. Detailed descriptions of the intervention processes, delivery, and implementation contribute to its reproducibility, which can be challenging in other studies [53–55]. Moreover, a brief systematic tool such as BI to deprescribe z-hypnotics in older patients can be useful to implement in clinical guidelines for physicians and in clinical practice. The BI is patient-centred, tailored to individual patient needs, and involves shared decision-making. The implication of this study is to enhance our understanding of z-hypnotics use related to multimorbidity, such as pain, cognition, and sleep in older patients, which are topics where current research is limited.

## Conclusion

We found no significant difference in inappropriate use between the BI and BAU, as both groups showed improvement. However, the study demonstrates that brief contact with patients regarding their z-hypnotics use, even as study-related information without intended intervention, can help them reduce or discontinue inappropriate usage in primary care. Older patients can stop inappropriate z-hypnotics use without significant worsening of sleep problems, pain, or cognitive function.

## Supplementary Information

Supplementary Material 1.

## Data Availability

All data generated or analysed during this study are included in this published article.
