# Supplementary material for: A brief intervention to discontinue inappropriate z-hypnotics use by older patients in primary care: a randomised controlled trial
Source: BMC Prim Care. 2026 Feb 13;27:97. doi: 10.1186/s12875-026-03212-w (PMC13005358; doi:10.1186/s12875-026-03212-w)
Supplement: Supplementary file 1 — Supplementary Material 1. [file 12875_2026_3212_MOESM1_ESM.docx]

**Supplement 1**

**Online questionnaire example (translated from Norwegian to English)**

Dear participant,

Below you will find a number of questions that we would like you to answer. There are no right or wrong answers; you choose the response that best fits your situation. When you are finished, you can press "submit."

ID Number:_____________________

**Gender**

Female

Name
Personal Identification Number (11 digits)
Gender

Female

Male

Other
What is your highest COMPLETED education?

Primary school (9-10 years)

Secondary school (12-13 years)

University and college (up to 3 years)

University and college (more than 3 years)
What is your "main language"?
The language you are most proficient in.

Which other languages do you speak?
Please specify other languages you are proficient in.

What is your household's total annual income before tax?
Including any benefits and social assistance.

Under 200,000 NOK

200,000 - 349,000 NOK

350,000 - 749,000 NOK

750,000 - 999,000 NOK

1 - 2 million NOK

Over 2 million NOK

Prefer not to say

Generer nytt svar

Kopier

Top of Form

Bottom of Form

Male

Name
Personal Identification Number (11 digits)
Gender

Female

Male

Other
What is your highest COMPLETED education?

Primary school (9-10 years)

Secondary school (12-13 years)

University and college (up to 3 years)

University and college (more than 3 years)
What is your "main language"?
The language you are most proficient in.

Which other languages do you speak?
Please specify other languages you are proficient in.

What is your household's total annual income before tax?
Including any benefits and social assistance.

Under 200,000 NOK

200,000 - 349,000 NOK

350,000 - 749,000 NOK

750,000 - 999,000 NOK

1 - 2 million NOK

Over 2 million NOK

Prefer not to say

Generer nytt svar

Kopier

Top of Form

Bottom of Form

Other

Name
Personal Identification Number (11 digits)
Gender

Female

Male

Other
What is your highest COMPLETED education?

Primary school (9-10 years)

Secondary school (12-13 years)

University and college (up to 3 years)

University and college (more than 3 years)
What is your "main language"?
The language you are most proficient in.

Which other languages do you speak?
Please specify other languages you are proficient in.

What is your household's total annual income before tax?
Including any benefits and social assistance.

Under 200,000 NOK

200,000 - 349,000 NOK

350,000 - 749,000 NOK

750,000 - 999,000 NOK

1 - 2 million NOK

Over 2 million NOK

Prefer not to say

Generer nytt svar

Kopier

Top of Form

Bottom of Form

**What is your highest COMPLETED education?**

Name
Personal Identification Number (11 digits)
Gender

Female

Male

Other
What is your highest COMPLETED education?

Primary school (9-10 years)

Secondary school (12-13 years)

University and college (up to 3 years)

University and college (more than 3 years)
What is your "main language"?
The language you are most proficient in.

Which other languages do you speak?
Please specify other languages you are proficient in.

What is your household's total annual income before tax?
Including any benefits and social assistance.

Under 200,000 NOK

200,000 - 349,000 NOK

350,000 - 749,000 NOK

750,000 - 999,000 NOK

1 - 2 million NOK

Over 2 million NOK

Prefer not to say

Generer nytt svar

Kopier

Top of Form

Bottom of Form

Primary school (9-10 years)

Name
Personal Identification Number (11 digits)
Gender

Female

Male

Other
What is your highest COMPLETED education?

Primary school (9-10 years)

Secondary school (12-13 years)

University and college (up to 3 years)

University and college (more than 3 years)
What is your "main language"?
The language you are most proficient in.

Which other languages do you speak?
Please specify other languages you are proficient in.

What is your household's total annual income before tax?
Including any benefits and social assistance.

Under 200,000 NOK

200,000 - 349,000 NOK

350,000 - 749,000 NOK

750,000 - 999,000 NOK

1 - 2 million NOK

Over 2 million NOK

Prefer not to say

Generer nytt svar

Kopier

Top of Form

Bottom of Form

Secondary school (12-13 years)

Name
Personal Identification Number (11 digits)
Gender

Female

Male

Other
What is your highest COMPLETED education?

Primary school (9-10 years)

Secondary school (12-13 years)

University and college (up to 3 years)

University and college (more than 3 years)
What is your "main language"?
The language you are most proficient in.

Which other languages do you speak?
Please specify other languages you are proficient in.

What is your household's total annual income before tax?
Including any benefits and social assistance.

Under 200,000 NOK

200,000 - 349,000 NOK

350,000 - 749,000 NOK

750,000 - 999,000 NOK

1 - 2 million NOK

Over 2 million NOK

Prefer not to say

Generer nytt svar

Kopier

Top of Form

Bottom of Form

University and college (up to 3 years)

NName
Personal Identification Number (11 digits)
Gender

Female

Male

Other
What is your highest COMPLETED education?

Primary school (9-10 years)

Secondary school (12-13 years)

University and college (up to 3 years)

University and college (more than 3 years)
What is your "main language"?
The language you are most proficient in.

Which other languages do you speak?
Please specify other languages you are proficient in.

What is your household's total annual income before tax?
Including any benefits and social assistance.

Under 200,000 NOK

200,000 - 349,000 NOK

350,000 - 749,000 NOK

750,000 - 999,000 NOK

1 - 2 million NOK

Over 2 million NOK

Prefer not to say

Generer nytt svar

Kopier

Top of Form

Bottom of Form

University and college (more than 3 years)

**What is your household's total annual income before tax?
*Including any benefits and social assistance.***

Under 200,000 NOK

NName
Personal Identification Number (11 digits)
Gender

Female

Male

Other
What is your highest COMPLETED education?

Primary school (9-10 years)

Secondary school (12-13 years)

University and college (up to 3 years)

University and college (more than 3 years)
What is your "main language"?
The language you are most proficient in.

Which other languages do you speak?
Please specify other languages you are proficient in.

What is your household's total annual income before tax?
Including any benefits and social assistance.

Under 200,000 NOK

200,000 - 349,000 NOK

350,000 - 749,000 NOK

750,000 - 999,000 NOK

1 - 2 million NOK

Over 2 million NOK

Prefer not to say

Generer nytt svar

Kopier

Top of Form

Bottom of Form

200,000 - 349,000 NOK

NName
Personal Identification Number (11 digits)
Gender

Female

Male

Other
What is your highest COMPLETED education?

Primary school (9-10 years)

Secondary school (12-13 years)

University and college (up to 3 years)

University and college (more than 3 years)
What is your "main language"?
The language you are most proficient in.

Which other languages do you speak?
Please specify other languages you are proficient in.

What is your household's total annual income before tax?
Including any benefits and social assistance.

Under 200,000 NOK

200,000 - 349,000 NOK

350,000 - 749,000 NOK

750,000 - 999,000 NOK

1 - 2 million NOK

Over 2 million NOK

Prefer not to say

Generer nytt svar

Kopier

Top of Form

Bottom of Form

NName
Personal Identification Number (11 digits)
Gender

Female

Male

Other
What is your highest COMPLETED education?

Primary school (9-10 years)

Secondary school (12-13 years)

University and college (up to 3 years)

University and college (more than 3 years)
What is your "main language"?
The language you are most proficient in.

Which other languages do you speak?
Please specify other languages you are proficient in.

What is your household's total annual income before tax?
Including any benefits and social assistance.

Under 200,000 NOK

200,000 - 349,000 NOK

350,000 - 749,000 NOK

750,000 - 999,000 NOK

1 - 2 million NOK

Over 2 million NOK

Prefer not to say

Generer nytt svar

Kopier

Top of Form

Bottom of Form

350,000 - 749,000 NOK

NName
Personal Identification Number (11 digits)
Gender

Female

Male

Other
What is your highest COMPLETED education?

Primary school (9-10 years)

Secondary school (12-13 years)

University and college (up to 3 years)

University and college (more than 3 years)
What is your "main language"?
The language you are most proficient in.

Which other languages do you speak?
Please specify other languages you are proficient in.

What is your household's total annual income before tax?
Including any benefits and social assistance.

Under 200,000 NOK

200,000 - 349,000 NOK

350,000 - 749,000 NOK

750,000 - 999,000 NOK

1 - 2 million NOK

Over 2 million NOK

Prefer not to say

Generer nytt svar

Kopier

Top of Form

Bottom of Form

750,000 - 999,000 NOK

NName
Personal Identification Number (11 digits)
Gender

Female

Male

Other
What is your highest COMPLETED education?

Primary school (9-10 years)

Secondary school (12-13 years)

University and college (up to 3 years)

University and college (more than 3 years)
What is your "main language"?
The language you are most proficient in.

Which other languages do you speak?
Please specify other languages you are proficient in.

What is your household's total annual income before tax?
Including any benefits and social assistance.

Under 200,000 NOK

200,000 - 349,000 NOK

350,000 - 749,000 NOK

750,000 - 999,000 NOK

1 - 2 million NOK

Over 2 million NOK

Prefer not to say

Generer nytt svar

Kopier

Top of Form

Bottom of Form

1 - 2 million NOK

NName
Personal Identification Number (11 digits)
Gender

Female

Male

Other
What is your highest COMPLETED education?

Primary school (9-10 years)

Secondary school (12-13 years)

University and college (up to 3 years)

University and college (more than 3 years)
What is your "main language"?
The language you are most proficient in.

Which other languages do you speak?
Please specify other languages you are proficient in.

What is your household's total annual income before tax?
Including any benefits and social assistance.

Under 200,000 NOK

200,000 - 349,000 NOK

350,000 - 749,000 NOK

750,000 - 999,000 NOK

1 - 2 million NOK

Over 2 million NOK

Prefer not to say

Generer nytt svar

Kopier

Top of Form

Bottom of Form

Over 2 million NOK

Prefer not to say

NName
Personal Identification Number (11 digits)
Gender

Female

Male

Other
What is your highest COMPLETED education?

Primary school (9-10 years)

Secondary school (12-13 years)

University and college (up to 3 years)

University and college (more than 3 years)
What is your "main language"?
The language you are most proficient in.

Which other languages do you speak?
Please specify other languages you are proficient in.

What is your household's total annual income before tax?
Including any benefits and social assistance.

Under 200,000 NOK

200,000 - 349,000 NOK

350,000 - 749,000 NOK

750,000 - 999,000 NOK

1 - 2 million NOK

Over 2 million NOK

Prefer not to say

Generer nytt svar

Kopier

Top of Form

Bottom of Form

“submit”
